# Genomics-based breeding in forest trees: are we there yet?

**DOI:** 10.1186/1753-6561-5-S7-I4

**Published:** 2011-09-13

**Authors:** David Neale

**Affiliations:** 1 University of California-Davis, USA

## 

Efforts to develop genetic marker based approaches to breeding forest trees began in the late 1980s. Approaches based on first generation of markers, allozymes, were not feasible due to the very limited number of markers (<50). The first DNA-based markers, RFLPs, brought more hope as moderately dense genetic maps could be constructed to scan the genome and map quantitative trait loci (QTLs). This approach was quite effective toward mapping QTLs in many forest tree species but the approach could not be brought to application in tree breeding due to low levels of linkage disequilibrium (LD) in forest tree breeding populations and recombination between flanking markers and QTLs with each generation. The next generation of DNA markers based of the polymerase chain reaction (PCR), RAPD, AFLP and SSR, did not solve the LD and recombination problem, even though more markers were available and throughput increased.

The situation began to change in the early 2000s with the availability of automated DNA sequencing technology and single nucleotide polymorphism (SNP) genetic markers. Now association studies could be performed where SNPs within candidate genes controlling complex traits could be identified and thus “solving” or minimizing the LD and recombination limitation. This approach to complex trait dissection has been widely applied in forest trees and the early approach of QTL mapping in segregating populations has been mostly abandoned. The association genetic approach has been used to find candidate gene SNPs associated to a broad array of quantitative traits of interest (wood properties, growth, abiotic stresses and disease resistance). However, like QTL mapping before, individual SNP x trait associations only account for a small proportion of the variation (generally less than 2-3% of the total phenotypic variance) and the total variation explained by all markers is generally less than 50%. In human genetics, this situation is called the “missing heritability” and there has been great debate over whether this problem can ever be solved in complex trait dissection in humans. In a few agricultural systems however, notably dairy cattle, researchers are now accounting for nearly all the heritable variation.

Efforts to realize genomics-based breeding in conifer tree improvement in the US have culminated under a collaborative research project funded by the USDA. The Conifer Translational Genomics Network Coordinated Agricultural Project (CTGN CAP) has successfully brought genomics-based breeding to application in the four major cooperative forest tree breeding programs in the US. The project has completed the basic research on allele discovery of economic traits in conifers, conducted the translational research, and provided education and training to tree breeders to realize this goal during the four-year project period (2007 to 2011). Genomics-based breeding can now be applied to the production of over 1.3 billion loblolly pine, slash pine and Douglas-fir seedlings planted annually in the US.

The CTGN CAP obtained high-density single nucleotide polymorphic (SNP) genotypes (over 7000 SNPs) on nearly 10,000 loblolly pine, slash pine, and Douglas-fir trees from the breeding cooperatives. These data were used to estimate molecular breeding values that can now be used to make selections as an alternative to breeding values based on mature traits that can take many years to the time of evaluation and can be expensive to measure in field tests. The CTGN CAP was the first to apply the advanced Illumina Infininium SNP-genotyping technology that is now being used in the breeding of most major crops such as corn, wheat, tomato, potato, and many more.

The CTGN CAP culminates a long-term investment by the USDA in this team of researchers and their work on the technological advancement of tree breeding in the US. USDA’s efforts began more than 20 years ago with funding of a series of grants under the National Research Initiative Plant Genome Program and continued under the CSREES IFAFS and AFRI programs. Recently, NIFA has awarded a grant to members of this team to conduct full genome sequencing in loblolly pine, sugar pine, and Douglas-fir. The full genome sequence will soon allow tree breeders to practice genomics-based breeding using genetic variation from the entire genome (genomic selection), and thus minimizing or even eliminating the missing heritability problem in forest trees. The partnership between USDA research programs and this team of researchers has resulted in a modernization of tree breeding technology in the US that will secure US competitiveness in the production of forest products.

